# An Easy and Efficient Method for Native and Immunoreactive *Echinococcus granulosus* Antigen 5 Enrichment from Hydatid Cyst Fluid

**DOI:** 10.1371/journal.pone.0104962

**Published:** 2014-08-13

**Authors:** Daniela Pagnozzi, Grazia Biosa, Maria Filippa Addis, Scilla Mastrandrea, Giovanna Masala, Sergio Uzzau

**Affiliations:** 1 Porto Conte Ricerche Srl, Tramariglio, Alghero, Sassari, Italy; 2 Centro Nazionale di Riferimento per l’Echinococcosi, IZS “G. Pegreffi”, Sassari, Italy; 3 Unità Operativa Complessa di Malattie Infettive, Azienda Ospedaliera Universitaria, Sassari, Italy; London School of Hygiene and Tropical Medicine, United Kingdom

## Abstract

**Background:**

Currently, the serodiagnosis of cystic echinococcosis relies mostly on crude *Echinococcus granulosus* hydatid cyst fluid as the antigen. Consequently, available immunodiagnostic tests lack standardization of the target antigen and, in turn, this is reflected on poor sensitivity and specificity of the serological diagnosis.

**Methodology/Principal Findings:**

Here, a chromatographic method enabling the generation of highly enriched Antigen 5 (Ag5) is described. The procedure is very easy, efficient and reproducible, since different hydatid cyst fluid (HCF) sources produced very similar chromatograms, notwithstanding the clearly evident and extreme heterogeneity of the starting material. In addition, the performance of the antigen preparation in immunological assays was preliminarily assessed by western immunoblotting and ELISA on a limited panel of cystic echinococcosis patients and healthy controls. Following western immunoblotting and ELISA experiments, a high reactivity of patient sera was seen, with unambiguous and highly specific results.

**Conclusions/Significance:**

The methods and results reported open interesting perspectives for the development of sensitive diagnostic tools to enable the timely and unambiguous detection of cystic echinococcosis antibodies in patient sera.

## Introduction

Cystic echinococcosis (CE) is caused by the larval form of *Echinococcus granulosus*. The life cycle of this cestode involves dogs and other canids as the definitive hosts, while intermediate hosts are usually cattle, sheep, goats and pigs [1]–[2]. However, due to the contamination of food and water sources with *E. granulosus* eggs shed with canid feces, man can become an accidental intermediate host and develop CE.

The greatest prevalence of CE in human and animal hosts has been recorded in the countries of the temperate zones, including parts of Eurasia, Australia, South America and Africa [3]. In most of these countries the disease has never been eradicated, exposing the population to the risk of reemergence in areas where CE was once believed to be controlled. In Bulgaria, following the reduction in control efforts, the incidence of CE in children peaked from 0.7 to 5.4/100000 between the 1970s and the mid-1990s. Similarly, in Wales the prevalence of infected dogs has more than doubled between 1993 (3.4%) and 2002 (8.1%) [4]. Outside the temperate zones, Italy is also currently considered a medium to high risk country for CE, with areas subjected to a significantly higher prevalence due to extensive sheep farming. In these high-risk areas, CE prevalence can represent a serious public health problem; as an example, the Island of Sardinia records a prevalence of 70–92.8% in sheep, 3–19% in dogs, 9.4% in cattle, 9.4–11.1% in pigs, and 1% in horses [5]–[6], while in the human population the annual regional average record (Hospital discharge rate) was 9.3 *per* 100000 inhabitants [7], with the occurrence of over 1000 cases requiring surgery each year in Italy [8]. Early diagnosis in humans is difficult, since the disease is typically asymptomatic for a long period after infection. Diagnosis is usually based on symptomatology, epidemiological data, and the combined use of radiologic imaging and immunodiagnostic techniques [9]. Imaging is mostly useful to assess the cyst stage and to differentiate echinococcal cyst from benign cysts, cavitary tuberculosis, mycoses, abscesses, and benign or malignant neoplasms [10]. On the other hand, serological tests might offer advantages over radiological procedures, including early diagnosis of infection, early treatment for a more effective chemotherapy, and close follow-up monitoring programs [11]. To date, however, commercially available immunoassays show unsatisfactory performances, possibly due to the poor quality of antigen preparations.

The hydatid cyst fluid (HCF), collected from infected animals, is the main source of antigens to be used for serological diagnosis. This liquid is a mixture containing a wide range of proteins of both parasite and host origin, the complexity and heterogeneity of which has already been highlighted by the proteomic characterization of HCF collected from sheep, cattle and humans [12]. The most abundant and immunogenic HCF proteins, despite the countless controversial publications about their specificity [1], [2], [13], [14], [15], [16], are Antigen 5 (Ag5) and Antigen B (AgB) [17]–[18]. Ag5 is an oligomeric thermolabile glycoprotein which migrates as 57 kDa and 67 kDa bands in sodium-dodecyl sulphate-polyacrylamide gel electrophoresis (SDS-PAGE) under non-reducing conditions, and as 38 kDa and 22 kDa bands under reducing conditions [19]. The immunodiagnostic power of the protein is thought to be mostly related to the 38 kDa subunit, which contains phosphorylcholine epitopes. These moieties have been demonstrated to play an important role both in cross-reactivity with sera of patients suffering from other diseases, and in immunoreactivity of Ag5 [16]. AgB is an oligomeric thermostable lipoprotein that dissociates, both under reducing and non reducing conditions, into 8/12, 16, and 20/24 kDa subunits, suggesting that it is composed of multimers of 8 kDa subunits [20]–[21].

During the last twenty years, many serological tests have been proposed, based mostly on crude HCF, Ag5, and AgB, but their diagnostic values have been proven to be limited in human hydatidosis. This is probably due to a combination of factors, including poor sensitivity and the lack of standardized antigen preparations. Moreover, it is worth noting that many published considerations refer to dated observations, often elaborated without the currently available advanced protein analysis facilities [22]–[25].

For all these reasons, an *ex novo* biochemical characterization of sheep HCF was performed by size exclusion chromatography, western immunoblotting, and mass spectrometry. We report here the set up of a reproducible size exclusion chromatography method that enables to obtain a highly enriched preparation of native Ag5. To evaluate the immunological reactivity of this antigen preparation, limited cohorts of sera from CE patient and healthy controls were evaluated against HCF crude antigen sample and against the Ag5 enriched chromatographic fraction. As preliminary results, we report that the enriched preparation of native Ag5 shows a very high and specific reactivity, both in western immunoblotting and in ELISA platforms, revealing promising perspectives for the development of sensitive immunodiagnostic tools.

## Methods

### Ethics statement

A written informed consent was obtained from patients at the time of sample collection. The study was approved by the ethics committee of the local health authority of Sassari (Comitato di Bioetica, ASL N. 1, Sassari), Prot N. 1123/L.

### Collection of sheep HCF and human serum samples

HCF crude samples were collected in two different Sardinian slaughterhouses (CE/IT2383M, Tula (SS) and CE/IT2078M, Lula (NU)), after a permission obtained from these slaughterhouses to use the animal parts from animals being processed as part of the normal work of the abattoir. Fluid was aspirated from liver and lung cysts found in infected sheep. The hydatid fluid was centrifuged at 1000×*g* at 4°C and the supernatant stored at −80°C. A total of 24 blood sera from CE patients and control subjects were obtained from a sample archive at the Istituto Zooprofilattico Sperimentale della Sardegna and were chosen by examining the clinical records available for each patient and control. All samples were leftovers from routine diagnostic procedures and had originally been collected from CE patients (n = 13) at the time of admission at the hospital. Negative sera were from Sardinian healthy blood donors (n = 6) or patients admitted at the hospital for other non-related diseases (n = 5). Diagnosis of CE had been performed by clinical examination and imaging according to standard clinical practice guidelines. Eight patients were surgically treated following admission to hospital and clinical diagnosis. CE was confirmed in all these cases by direct inspection of parasite material.

### SDS-PAGE

Four different HCF samples (from two lung and two liver cysts) were thawed and centrifuged at 12000×*g* at 4°C. The supernatants were then five-fold concentrated and desalted by ultrafiltration on centrifugal filter devices with a molecular mass cut-off of 10 kDa (Microcon YM-10, Millipore). For each sample, total protein concentration, evaluated by the BCA assay (Pierce, Thermo Scientific), was quantified before and after ultrafiltration. Finally, Laemmli buffer with or without 400 mM dithiothreitol (DTT) as a reducing agent was added to 20 µg of each sample [26], and subjected to SDS-PAGE on 15% acrylamide gels using the Mini-Protean system (Bio-Rad, Hercules, CA, USA).

After BCA assay, Laemmli buffer with or without 400 mM DTT was added to four different aliquots (500 ng, 1 µg and 2 µg) of size exclusion Ag5 fraction, and the samples were subjected to SDS-PAGE on 4–10% acrylamide gel. Gel was stained by silver nitrate procedures [27]–[28].

### Western Immunoblotting

Western immunoblotting was performed as described previously [29], with minor modifications. Briefly, proteins separated by SDS-PAGE (on 15% or 4–10% acrylamide gels) were electrically transferred onto nitrocellulose membranes and blocked in phosphate buffered saline, pH 7.4, 0.05% Tween 20 (PBS–T), containing 5% skim milk for 2 h. Membranes were then incubated with sera (1∶200 in PBS-T, 2% skim milk) for 1 h, either directly on membranes containing antigen preparations loaded in individual wells of 10-well gels, or by using a multiscreen apparatus (Bio-Rad) on membranes containing antigen preparations loaded in single-well gels. After washing, membranes were incubated with horseradish peroxidase conjugated anti-human IgG (Sigma) diluted 1∶500000 in 2% skim milk in PBS-T, developed with ECL substrate (Sigma), and digitized with VERSA DOC 4000 MP (Biorad).

### Antigen 5 preparation

Ag5 fraction was obtained by Fast Protein Liquid Chromatography (FPLC) procedures, performed on an AKTA Explorer 10 system (GE Healthcare). Aliquots of 5 mL of 5 sheep HCF were desalted by HiPrep 26/10 column (GE Healthcare), lyophilized, solubilized in 500 µL of 50 mM phosphate buffer at pH 7.6, containing 150 mM NaCl (running buffer) and filtered at 12,000×*g* on PVDF 0.22 µm filters (Ultrafree-MC Centrifugal Filter Units, Millipore). Size exclusion chromatography fractionations were then performed on a Superdex-200 column (10/300 GL, GE Healthcare). Runs were performed at room temperature at a flow rate of 1 mL/min. Column was calibrated, under the same conditions, with standard proteins (thyroglobulin, apoferritin, beta-amylase, BSA and carbonic anhydrase, Sigma Aldrich).

### *In situ* hydrolysis and Mass Spectrometry analysis of gel bands

Gel bands (selected ones or the whole lanes) were cut and subjected to *in* *situ* hydrolysis as described previously [30]; briefly, gel slices were destained, reduced in 50 mM NH_4_HCO_3_ buffer with 10 mM DTT at 56°C and then carbamidomethylated in 50 mM NH_4_HCO_3_ buffer with 55 mM iodoacetamide at room temperature in the dark. Tryptic digestion was performed at 37°C overnight using variable amount (50–100 ng) of trypsin per gel slice.

Peptide mixtures obtained by selected bands from HCF were analyzed by LC-MS/MS on a XCT Ultra 6340 ion trap equipped with a 1200 HPLC system and a chip cube (Agilent Technologies, Palo Alto, CA). After loading, peptides were concentrated and desalted at 4 µl/min on a 40 nL enrichment column, with 0.2% formic acid and then fractionated on a C18 reverse-phase (75 µm×43 mm, Agilent Technologies Chip) at a flow rate of 300 nl/min, with a linear gradient of eluent B (0.2% formic acid in 95% acetonitrile) in A (0.2% formic acid in 5% acetonitrile) from 3 to 60% in 20 min. MS method parameters were as follows: Capillary voltage 1730 V. Trap ICC smart target was 300000 units and maximal accumulation time was 100 ms. MS/MS was operated at a fragmentation amplitude of 1.3 V, and threshold ABS was 6000 units. Scan speed was set in “standard-enhanced” mode at 8100 (m/z)sec^−1^ for MS and “ultra scan” mode at 26000 (m/z)sec^−1^ for MS/MS scans. Peptide analysis was performed scanning from m/z 250 to m/z 2200 in AutoMS (n) precursor selection mode of the three most intense ions (fragmentation mass range from 100 to 2200 m/z). Dynamic exclusion was used to acquire a more complete survey of the peptides by automatic recognition and temporary exclusion (0.15 min) of ions from which definitive mass spectral data had previously acquired. Helium was used as the collision gas. Data Analysis software (6300 Series Ion Trap LCMS), provided by the manufacturer, was used to analyze raw MS and MS/MS spectra and to generate peak lists, as MGF files.

The whole lane analysis (GeLC-MS/MS) of Ag5 fraction from size exclusion chromatography was performed on a Q-TOF hybrid mass spectrometer equipped with a nano lock Z-spray source and coupled on-line with a nanoAcquity chromatography system (Waters). The peptide mixture was concentrated and washed onto a Trap column (Symmetry 100 Å, 5 µm, C18, 180 µm×20 mm, Waters) using 0.2% formic acid, and fractionated onto a C18 RP column (NanoAcquity UPLC 1.7 µm BEH130 C18 75 µm×150 mm, Waters) at a flow rate of 250 nL/min. The samples were fractionated using a linear gradient of eluent B (0.2% formic acid in 95% ACN) in eluent A (0.2% formic acid) from 0.5 to 50% B in 46 minutes. The mass spectrometer was set up in a data-dependent MS/MS mode where a full-scan spectrum was followed by tandem mass spectra, selecting peptide ions as the three most intense peaks of the previous scan. MS method parameters were as follows: Capillary voltage 2000 V, Scan time 0.5 sec in MS and 1 sec in MSMS. Peptide analysis was performed scanning from m/z 300 to m/z 1600 in MS and from 100 to 2000 m/z in MSMS. Dynamic exclusion was used to acquire a more complete survey of the peptides by automatic recognition and temporary exclusion (10 sec) of ions from which definitive mass spectral data had previously acquired. Argon was used as the collision gas, and collision energy depending on mass and charge of the precursor ion was applied. ProteinLynx software (Version 2.2.5), was used for analysis of raw MS and MS/MS spectra and the production of the pkl lists.

Both the peak lists from Q-TOF analysis, converted into a MGF file, and the MGF files from Ion Trap mass spectrometer were analyzed by Proteome Discoverer software (version 1.4; Thermo Scientific, Bremen, Germany) using Sequest-HT as search engine for protein identification, according to the following criteria: Database UniprotKB (release 2014_02), taxonomy Mammalia integrated with *Echinococcus granulosus* [31]–[32], enzyme trypsin, precursor mass tolerance 30 ppm and fragment mass tolerance 0.3 Da for Q-TOF analysis, mass tolerance 200 ppm and fragment mass tolerance 0.6 Da for Ion Trap analysis, cysteine carbamidomethylation as static modification and N-terminal glutamine conversion to pyroglutamic acid and methionine oxidation as dynamic modifications. The percolator algorithm was used for protein significance (p-value<0.01) and for peptide validation (peptide confidence: q-value<0.05). Only rank 1 peptides and only proteins identified with at least two peptides and two spectral counts were considered.

### Data analysis

Protein abundance was expressed by means of the normalized spectral abundance factor (NSAF). NSAF was calculated as follows: 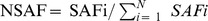, where subscript i denotes a protein identity and N is the total number of proteins, while SAF is a protein spectral abundance factor that is defined as the protein spectral counts divided by its length (number of residues or molecular weight). In this approach, the spectral counts of each protein were divided by its length and normalized to the total sum of spectral counts/length in a given analysis [33].

### ELISA

For indirect ELISA tests, microplates (Nunc-Maxisorp Immunoplate) were coated with Ag5 fraction by depositing in each well 100 µL of a 100 ng/mL antigen solution in PBS. Plates were then incubated at 37°C until completely dried [34], washed with 0.05% Tween in PBS (PBS-T), and incubated for 1 h at 37°C in blocking solution (5% bovine serum albumin (BSA) in PBS-T). After washing with PBS-T, sera were added at 1∶500 dilution in 2% BSA in PBS-T and incubated at 37°C for 1 hour. Secondary antibody (horseradish peroxidase conjugated anti-human IgG, Sigma) was diluted 1∶100000 in 2% BSA in PBS-T and incubated at 37°C for 1 hour. Finally, the substrate (3.3′, 5.5′-Tetramethyl-benzidine Liquid Substrate, Supersensitive, Sigma) was added. Wells were washed five times with PBS-T between the application of sera and the secondary antibody, as well as between the application of the secondary antibody and the substrate. The absorbance was read at 620 nm at 15′, 30′ and 1 hour of incubation on a Tecan Sunrise microplate reader. The readings were taken at different time points to find out the best performing incubation time, and then only 60 min incubation times were considered for calculations. All sera were tested in duplicate, and net absorbances were calculated by subtracting the reactivity of the secondary antibody and substrate alone. Threshold value was established as the mean + 2SD of all the negative controls. All patient sera were also tested in parallel with four commercially available ELISA kits for CE diagnosis according to the user instructions provided (Euroimmun, DRG, MP biomedicals, Serion). All net absorbance values were used for statistical calculations (Student’s t-test) and for plotting the results obtained with sera from CE positive patients and controls using Microsoft Excel (Microsoft, Redmond, WA).

## Results

### SDS-PAGE of sheep HCF

**Figure 1:**
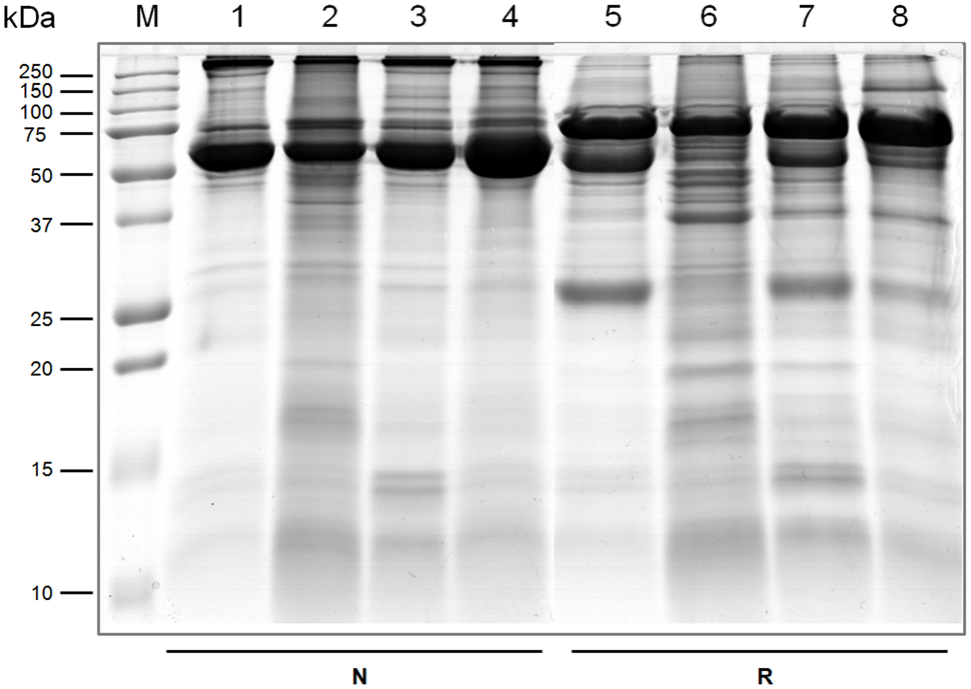
SDS-PAGE separation of sheep HCF. Two lung (lanes 1–2, 5–6) and two liver (lanes 3–4, 7–8) HCF samples were separated under non-reducing (N, lanes 1–4) and reducing conditions (R, lanes 5–8) by SDS-PAGE on 15% acrylamide gels. Each sample was loaded after concentration and desalting by ultrafiltration. Due to the different starting protein concentrations, different volumes of each sample, ranging from 7.5 µL to 17.5 µL, where used to reach loads of 20 µg/lane. Coomassie Blue staining was performed. Lane M: molecular weight markers.

A total of 4 different HCF samples collected from hydatid cysts, two localized in the lung and two in the liver of infected sheep, were concentrated and desalted by ultrafiltration and then analyzed by SDS-PAGE under non-reducing and reducing conditions (Figure 1). Protein concentration, quantified before and after ultrafiltration, revealed heterogeneous values among the four samples. Indeed, even though 20 µg of sample was loaded in each lane, this protein amount was reached by using different volumes for the 4 fluids. An extreme variability was observed not only in HCF protein concentration, but also in its protein composition, as clearly evident when comparing the different band distribution and their relative abundance (lanes 1–4 as well as lanes 5–8) probably due to presence of both host and parasite proteins in variable amounts. Therefore, a strategy for fractionation and characterization of the fractions was devised.

### Western immunoblotting on sheep cystic fluid and LC-MS/MS characterization of reactive bands

**Figure 2:**
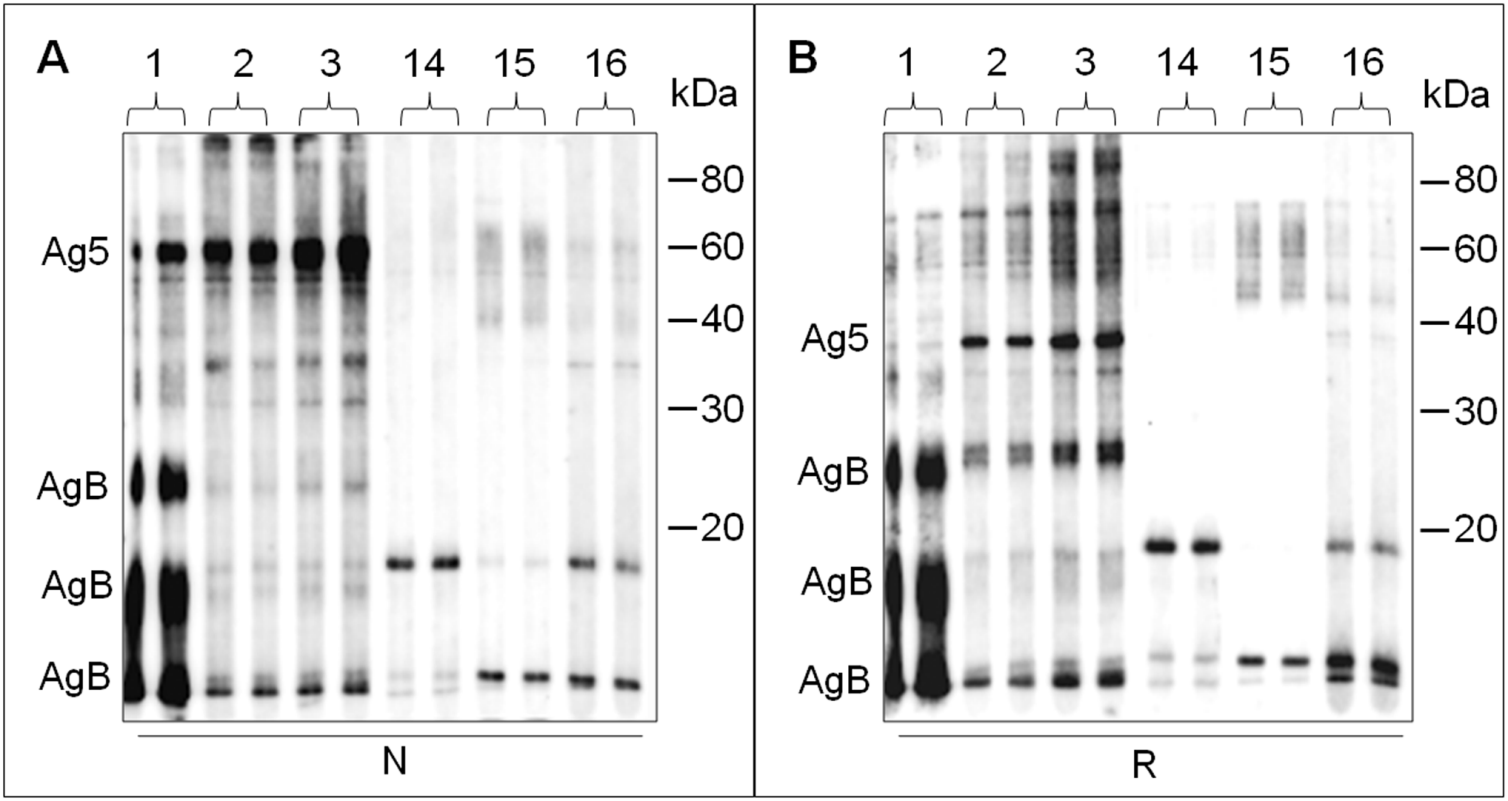
Western immunoblotting of human sera against crude HCF. Membranes containing HCF antigen preparations, loaded in single-well gels, were incubated with blood sera from CE patients (1–3) and control subjects (14–16) by using a multiscreen apparatus. Panel A and B show sera reactivity against non-reduced and reduced (N and R) HCF, respectively. The approximate positions of AgB and Ag5 *E. granulosus* antigens are indicated on the left. Positive sera 2 and 3 do not seem to recognize the typical ladder-like pattern of AgB in both conditions. On the other hand, serum 1 recognizes only weakly the 38 kDa band putatively arising from Ag5 reduction (panel B). Moreover, control sera produce non specific responses toward some protein components. Sera were tested in duplicate in at least three separate experiments. Molecular weight references are indicated on the right.

In order to characterize the reactivity and specificity of antibodies in sera of CE patients, a pool of crude HCF antigen preparation was subjected to western blotting analysis on a multiscreen apparatus. HCF was run under non-reducing and reducing conditions and resulting membranes were both incubated with a collection of 24 sera, 13 of which were from CE patients and the other 11 were from healthy controls. As an example, Figure 2 shows the results obtained with 3 CE patients and 3 healthy controls. Reactivity against numerous protein bands was observed for CE patients (1–3), together with few bands also for control sera (14–16). Moreover, patients 1–3 showed different behaviours, both in non reducing and reducing conditions. More in detail, only serum 1 recognized a band triplet at about 8-16-24 kDa in both the experimental conditions used, while showing a faint signal for a 38 kDa band in reducing conditions.

**Figure 3:**
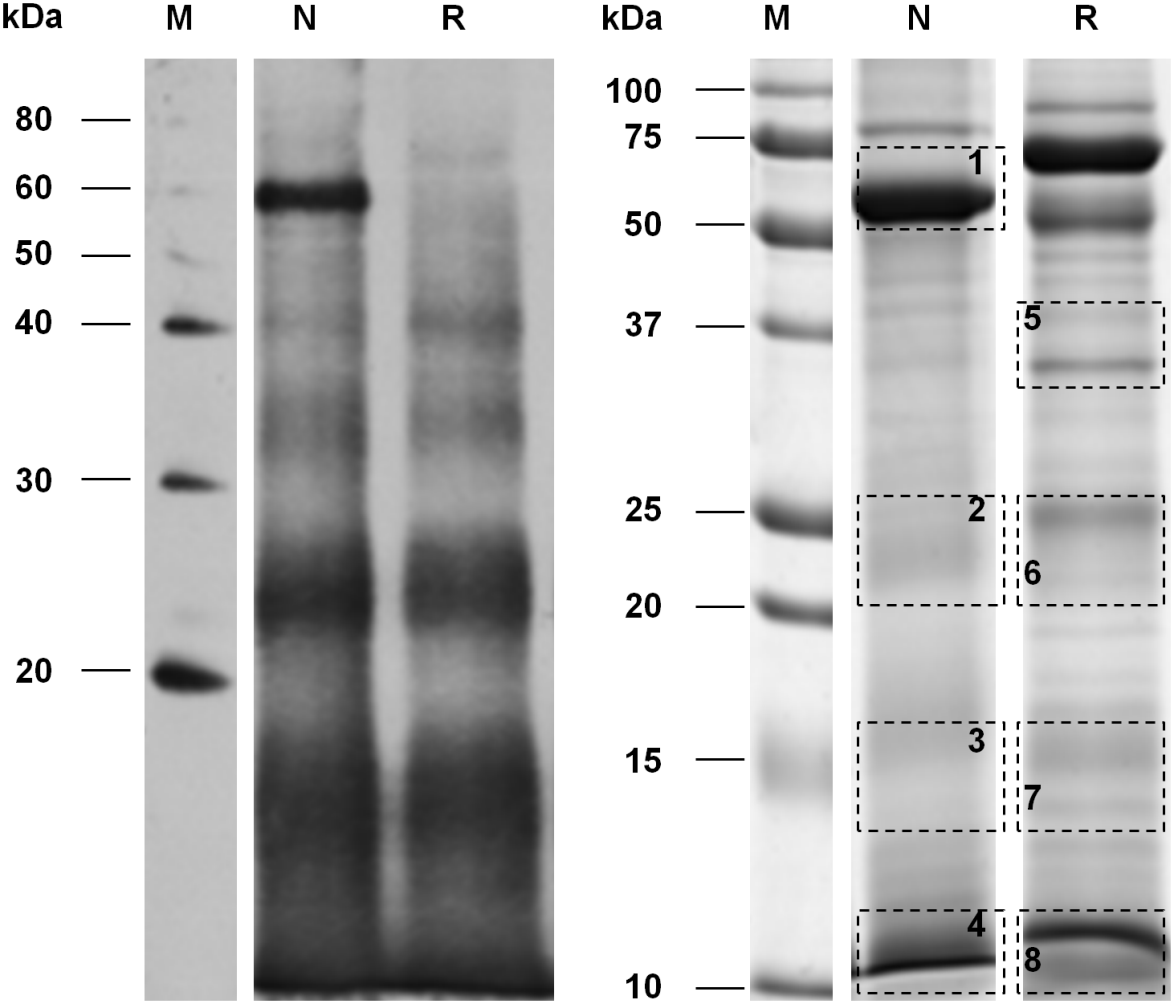
Immunoreactive bands subjected to mass spectrometry analysis. HCF was separated by SDS-PAGE and the resulting gel was cut in two parts, one of them subjected to western blot experiment with serum 1, the other part stained and further processed for protein identification Left: Immunoreactive profile observed by western immunoblotting on non-reduced (N) and reduced (R) HCF samples. Right: reactive bands excised from the SDS-PAGE gel and subjected to LC-MS/MS identification. M: molecular weight markers.

To shed light on the identity of the proteins which gave reactivity in CE patients and to gather information on the HCF composition, an SDS-PAGE of HCF was performed and the gel was cut in two parts; one half was subjected to a western blot experiment with serum 1, whilst the second half was Coomassie stained and used for protein identification by tryptic digestion followed by tandem mass spectrometry (LC-MS/MS) analysis (Figure 3). More in detail, by comparing the two parts of the gel, the areas corresponding to the reactive bands on the western blot image were excised from the stained gel and subjected to identification. The complete list of protein identities is reported in Table 1, and all the details about the identifications are reported in Table S1. As a result, the strongly reactive bands observed at about 8-16-24 kDa only with serum 1 in both conditions were identified as AgB, with its typical ladder-like pattern (bands 2, 3, 4, 6, 7, 8 Figure 3), while the 38 kDa band seen in reducing conditions for sera 2 and 3 was identified as Ag5 (band 5 Figure 3). The prominent reactive band seen at 60 kDa in non-reducing conditions for all three CE patient sera (band 1 Figure 3) was identified as Ag5. In general, therefore, CE patient sera displayed a homogeneous reactivity for the non-reduced Ag5 60 kDa band and a heterogeneous reactivity for AgB and for the reduced Ag5 38 kDa band. Control sera displayed a light reactivity, most likely non-specific and directed against both parasite and host proteins present in the crude HCF preparation.

**Table 1:** List of identified proteins from selected SDS-PAGE bands of Figure 1.

Band	Protein name	Species	Acc. No.^a^
**NON REDUCED SAMPLE**			
1	Ag5	*E. granulosus*	I1WXU1
	Serum albumin	*O. aries*	P14639
2	Antigen B 4/1	*E. granulosus*	D1MH02
	Phosphatidylethanolamine-binding protein	*B. taurus*	P13696
3	Antigen B subunit 4	*E. granulosus*	Q6UZD9
4	Antigen B 4/1	*E. granulosus*	D1MH02
**REDUCED SAMPLE**			
5	Ag5	*E. granulosus*	I1WXU1
6	Antigen B 4/1	*E. granulosus*	D1MH02
7	22 kDa antigen 5	*E. granulosus*	D6R8R1
	Phosphoenolpyruvate carboxykinase	*E. granulosus*	I3NX81
	Antigen B 4/1	*E. granulosus*	D1MH02
8	Antigen B subunit 2	*E. granulosus*	Q6EJE1

a UniprotKB accession number.

### Size exclusion chromatography of sheep HCF and fraction characterization

**Figure 4:**
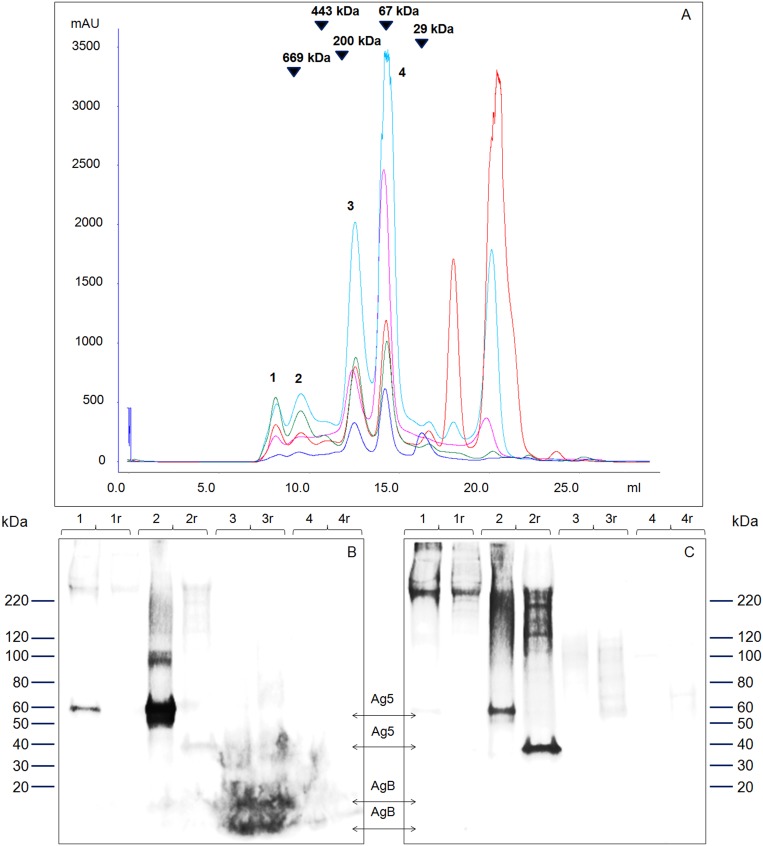
Size exclusion chromatography of sheep HCF. A. Overlay of five HCF chromatograms showing the highly reproducible profile achievable despite the heterogeneity of the starting samples. Aliquots of sheep HCF were desalted and loaded on a Superdex-200 column. Size calibration marks, obtained from runs of standard proteins (thyroglobulin, apoferritin, beta-amylase, BSA and carbonic anhydrase), are indicated by arrows in the chromatogram. B and C: the numbered peak fractions (1–4) were collected and analyzed by western immunoblotting, after SDS-PAGE on 4–10% acrylamide gels, under non reducing and reducing (r) conditions. Two patient sera (serum 1, panel B, and serum 2, panel C, respectively) were tested against all the fractions. Arrows indicate the position of Ag5 and AgB.

The strongest and immunodominant reactivity observed in patient sera was directed against native, non-denatured Ag5. Hence, a separation protocol by size exclusion chromatography was developed and optimized in order to attain an Ag5 enriched preparation under native conditions (Figure 4A). The main chromatographic fractions generated from all HCF samples, indicated with numbers from 1 to 4, were collected and analyzed by SDS-PAGE under non-reducing and reducing conditions, and probed by immunoblotting with two representative patient sera (Figure 4B and 4C). As a result, western blot analysis showed that Ag5 was mainly contained in fraction 2, whilst AgB was mainly eluted in fraction 3; moreover, the reduced Ag5 38 kDa band was only weakly recognized by serum 1 (Figure 4B, lane 2r); finally, AgB was recognized only by serum 1 in fraction 3 (Figure 4B, lanes 3-3r).

As clearly evident in Figure 4A, the Ag5 preparation procedure is highly reproducible, despite the extreme heterogeneity seen in the protein abundance profile of different HCF samples, as already highlighted by SDS-PAGE (Figure 1). Indeed, the elution volume of the Ag5 fraction was consistently at 9.50 mL, for all the five HCF runs, demonstrating the robustness and reproducibility of this procedure.

**Figure 5:**
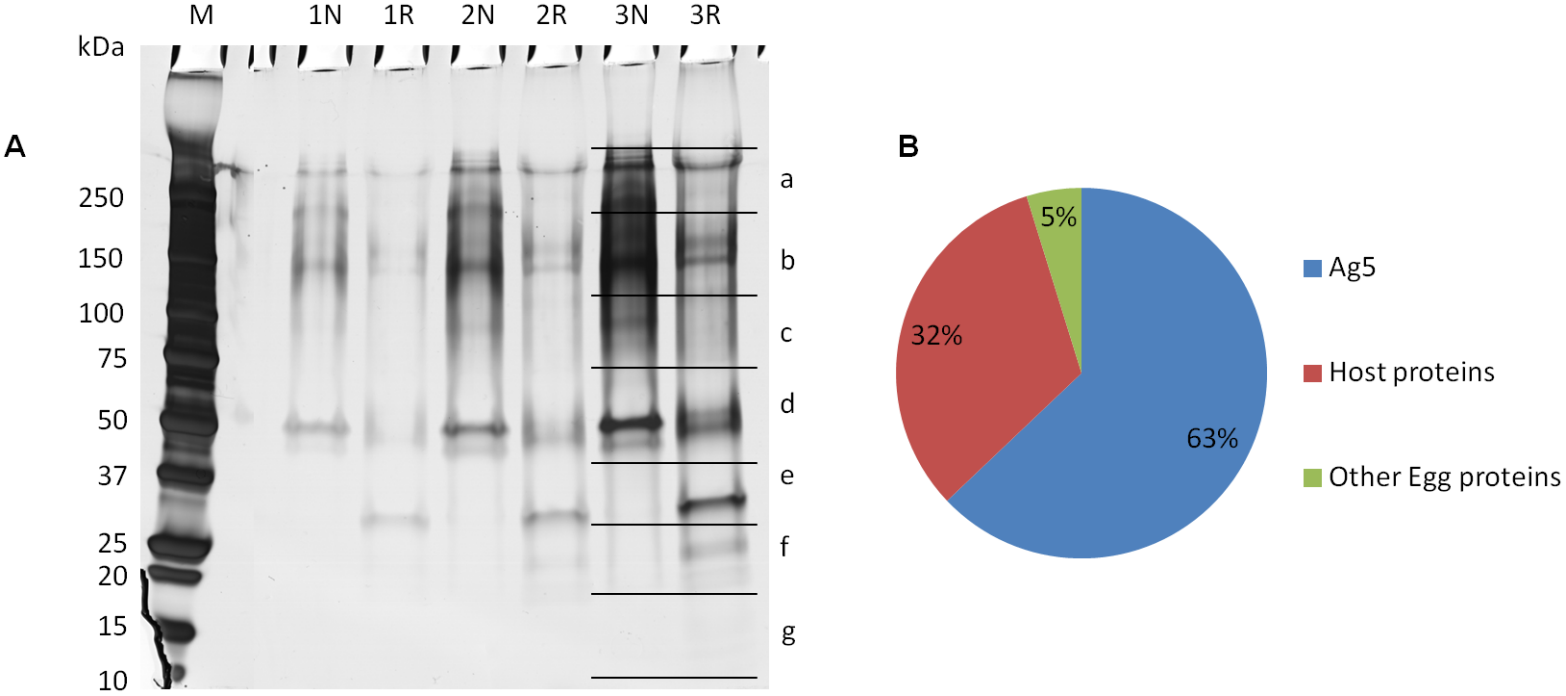
SDS-PAGE and relative protein abundance of fraction 2 from size exclusion chromatography of sheep HCF. A: Laemmli buffer with (R) or without 400 mM DTT (N) was added to aliquots of 500 ng (lanes 1N-1R), 1 µg (lanes 2N-2R) and 2 µg (lanes 3N-3R) of size exclusion chromatography fraction 2, and subjected to SDS-PAGE on 4–10% acrylamide gel. Lane M: molecular weight markers. Gel was silver stained and then analyzed by GeLC-MS/MS, by cutting lanes 3N and 3R in 7 bands (a–g) each. B: pie chart representing the relative protein abundance of fraction 2 from GeLC-MS/MS analysis of lane 3R. According to label free quantitation, Ag5 represents the most abundant component of the fraction, reaching about 63% of the total content.

According to the standard curve performed on the size exclusion column, the apparent native molecular weight of Ag5 was of at least 600 kDa, corresponding to an oligomeric structure of about ten units, or to a native protein complex involving Ag5. In order to investigate this issue, an SDS-PAGE of fraction 2 was run and silver stained (Figure 5A). Both whole lanes corresponding to 2 µg of non-reduced and reduced fraction 2 (lanes 3N-3R) were analyzed by GeLC-MS/MS and the identifications revealed the fraction 2 composition (Table 2 and Table S2). Adding to Ag5, the high molecular weight bands observed both in western blots in Figure 4 (lane 2 and 2r) and in electrophoresis in Figure 5A (bands a and b in lanes 3N and 3R) could be reasonably attributed to the basement membrane specific heparan sulfate protein (identified in bands 3Na and 3Ra), peroxidasin (identified in bands 3Na, 3Nb and 3Rb) and neurogenic locus notch protein (identified in bands 3Na and 3Rb), according to MS identifications of single bands (Table 2). However, it cannot be excluded that the observed immunoreactivity is still due to the Ag5 migrating in these higher molecular weight bands.

**Table 2:** List of identified proteins from GeLC-MS/MS analysis of size exclusion chromatography fraction 2.

Protein name	Species	Acc. No.^a^	Bands^b^	%^c^
**NON REDUCED**				
Ag5	*E. granulosus*	I1WXU1	3Na, 3Nb, 3Nc, 3Nd, 3Ne, 3Nf	74.6
Serum albumin	*O. aries*	P14639	3Na, 3Nd, 3Ne, 3Ng	17.1
Basement membrane specific heparan sulfate	*E. granulosus*	U6JHS5	3Na	0.5
Neurogenic locus notch protein	*E. granulosus*	U6JDN7	3Na	0.9
Fibronectin	*B. taurus*	P07589	3Na	0.7
Peroxidasin	*E. granulosus*	U6JLH4	3Na, 3Nb	1.3
Lysosomal alpha glucosidase	*E. granulosus*	U6JQ59	3Na, 3Nb, 3Nc	2.2
Alpha-2-macroglobulin	*B. taurus*	Q7SIH1	3Na	0.6
Cathepsin D lysosomal aspartyl protease	*E. granulosus*	U6J5K4	3Nc, 3Nd	2.0
Laminin	*E. granulosus*	U6JF91	3Nc	0.3
**REDUCED**				
Ag5	*E. granulosus*	I1WXU1	3Rb, 3Rc, 3Rd, 3Re, 3Rf, 3Rg	63.0
Serum albumin	*O. aries*	P14639	3Ra, 3Rb, 3Rc, 3Rd, 3Re, 3Rf, 3Rg	31.5
Peroxidasin	*E. granulosus*	U6JLH4	3Rb, 3Rf, 3Rg	2.2
Neurogenic locus notch protein	*E. granulosus*	U6JDN7	3Rb, 3Re	0.8
Basement membrane specific heparan sulfate	*E. granulosus*	U6JHS5	3Ra	0.2
Lysosomal alpha glucosidase	*E. granulosus*	U6JQ59	3Rc, 3Rf, 3Rg	0.9
Alpha-2-macroglobulin	*B. taurus*	Q7SIH1	3Ra, 3Rb, 3Rc	0.5
Fibronectin	*B. taurus*	P07589	3Rb	0.2
Laminin	*E. granulosus*	U6JF91	3Rd	0.5

a UniprotKB accession number.

b Bands in which the protein was identified.

c Percentage content according to label free quantitation approach.

By using this data, label free quantitation with the spectral count approach applied to lane 3R allowed also to provide a rough estimate of the relative protein content in fraction 2. As described in Figure 5B, after size exclusion chromatography the Ag5 percentage content in the analyzed fraction was estimated to be 63%, while the remaining proteins were 32% host proteins and 5% other *E. granulosus* proteins.

### Western blotting on enriched native Ag5 fraction

**Figure 6:**
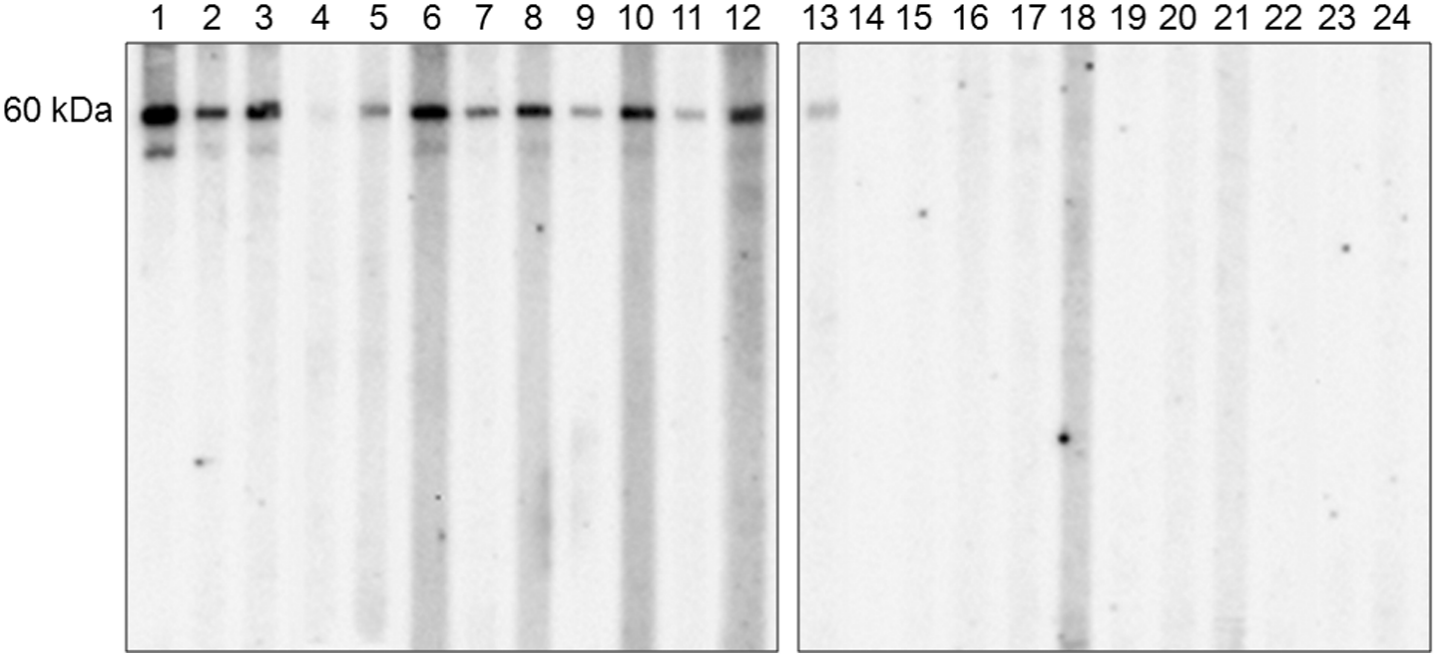
Western immunoblotting of human sera against Ag5 enriched preparation. Fraction 2 from size exclusion chromatography of HCF was used as antigen in western blotting experiments under non-reducing conditions. Sera from CE patients (1–13) and control subjects (14–24) were tested against a total of 300 ng of protein sample loaded in each single-well gel; this amount corresponds approximately to about 10 ng of proteins per multiscreen slot. All CE patients sera react against the Ag5 protein band, although with a variable intensity probably depending on the antibody titer of each serum. Moreover, control sera do not give any non-specific response. All sera were tested in at least three separate experiments. The molecular weight region of Ag5 (60 kDa) is indicated on the left.

To assess its diagnostic value, the chromatographic Ag5 preparation was assayed by immunoblotting on a multiscreen apparatus, with a collection of 24 human sera available in this study (Figure 6). An unambiguous reactivity of patient sera against the non-reduced, enriched Ag5 fraction was observed, when compared to the heterogeneous results seen with crude HCF (Figure 2); moreover, a significant reduction in nonspecific signals was detected with control sera.

### Performance of the enriched native Ag5 preparation on an ELISA platform and comparison with four commercial ELISA assays

**Figure 7:**
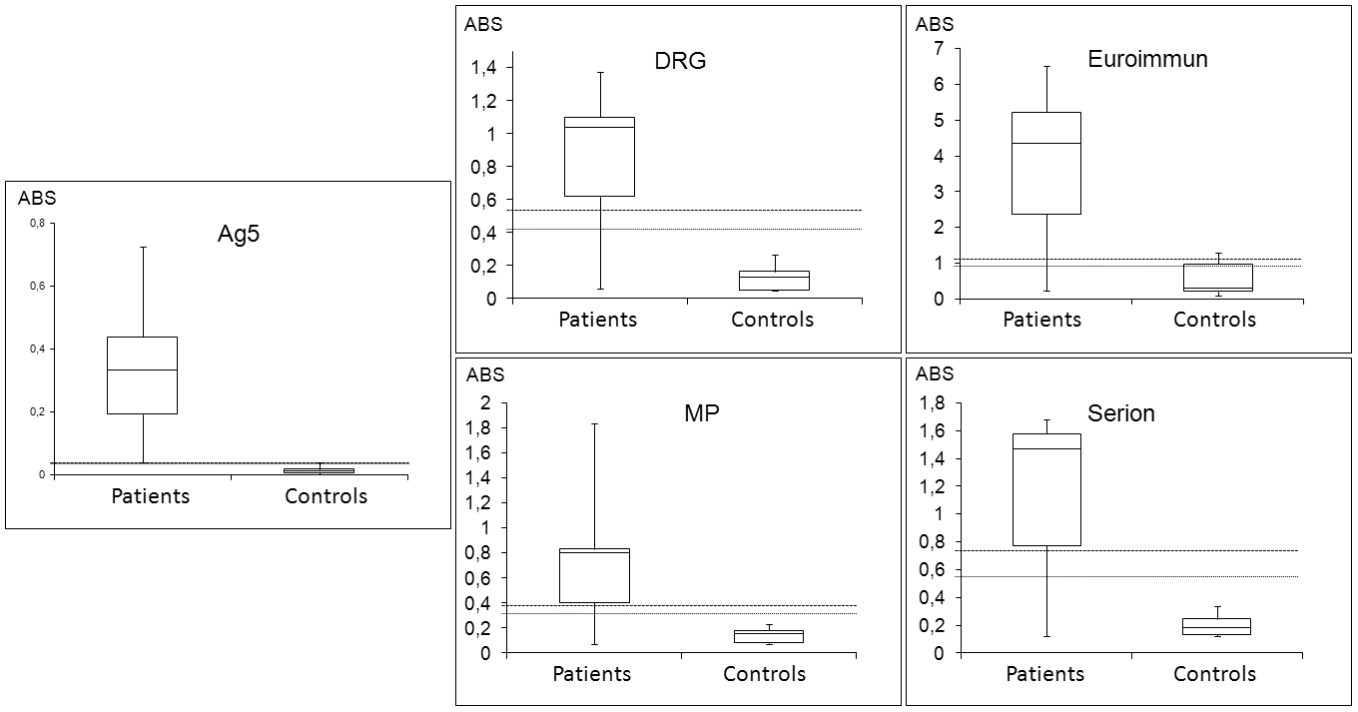
Comparative evaluation of Ag5 and commercial ELISA kits. Boxplots summarizing the absorbance values obtained with the Ag5 preparation and commercial ELISA kits on CE patients and control subjects. Patient and control sera are plotted according to the clinical status. The dashed and dotted lines indicate the upper and lower cutoff values, respectively. ABS: absorbance at the wavelength required for each assay. Differences in absorbance between the patient and control groups were statistically significant for all the assays (P<0.001).

The suitability of the enriched Ag5 preparation to the ELISA format was then evaluated on all patient and control sera (Ag5 ELISA). As a comparison, the same sera were also tested with four ELISA kits currently used for serological evaluation of CE patients (DRG, Serion, Euroimmun, and MP). Table 3 summarizes the results obtained, and presents the specificity and sensitivity values calculated for each test, while Table S3 and Figure 7 illustrate absorbance values obtained for positive and control sera as values and as boxplots, respectively. The data distribution reported in the boxplots and the summary table demonstrate a better separation of the patient and control groups with the Ag5 enriched antigen, showing a greater sensitivity when compared to commercially available ELISAs (Table 3), although the number of sera is too limited to draw statistically supported conclusions. The Ag5 ELISA enabled the detection of antibodies against *E. granulosus* in all clinically positive patients, while patients 4 and 5 tested negative with all the HCF-based assays. In addition, patient 9 tested negative with three out of four commercial assays. Specificity was also high when compared to the most specific commercial assay, indicating that sensitivity can be increased without incurring in detection of false positive results. Because of the unavailability of sera, specificity was not evaluated for patients with different parasitoses.

**Table 3:** Summary of the ELISA results obtained with the Ag5 enriched antigen test and four commercial kits.

Patient	Clinical Diagnosis	Cyst n° and localization	ELISA				
			Ag5	DRG	Euroimmun	MP	Serion
1	+	1 liver	+	+	+	+	+
2	+	1 lung	+	+	+	+	+
3	+	1 lung	+	+	+	+	+
4	+	1 liver, 1 lung	+	−	−	−	−
5	+	1 liver	+	−	−	−	−
6	+	2 liver	+	+	+	+	+
7	+	1 lung, 1 medullary	+	+	+	+	+
8	+	1 liver, 2 lung	+	+	+	+	+
9	+	1 liver, 1 lung	+	−	+	−	−
10	+	1 liver	+	+	+	+	+
11	+	5 lung	+	+	+	+	+
12	+	multiple scattered	+	+	+	+	+
13	+	1 lung	+	+	+	+	+
14	−		−	−	+	−	−
15	−		−	−	−	−	−
16	−		−	−	−	−	−
17	−		−	−	−	−	−
18	−		−	−	+	−	−
19	−		−	−	−	−	−
20	−		−	−	−	−	−
21	−		−	−	+	−	−
22	−		−	−	−	−	−
23	−		−	−	+	−	−
24	−		−	−	−	−	−
% Sensitivity			100	77	85	77	77
% Specificity			100	100	64	100	100

## Discussion

Despite almost fifty years of active investigation, immunodiagnosis of CE [35] is still a challenge. Many assay platforms have been devised, including indirect hemagglutination, latex agglutination, immunofluorescence, immunoelectrophoresis, western immunoblotting, and ELISA [36]–[40], based on various antigen preparations. In the last fifteen years, purified native, recombinant, or synthetic proteins and peptides have been increasingly evaluated, ranging from the well known Ag5 and AgB [18], [41], to the less popular malate dehydrogenase [15], P29 [42], thioredoxin peroxidase [43], and the 89 kDa together with the 74 kDa protein described by Carmena [44], among others.

Currently, commercially available serological kits are mostly based on ELISA and use crude HCF as target antigen because of its ease of preparation, as well as for its availability in endemic areas. However, in its crude form generally obtained from cysts developed in infected sheep, this fluid contains a complex mixture of host proteins, mainly represented by blood serum proteins, together with proteins released by *E. granulosus*. In this work, SDS-PAGE and size exclusion chromatography (Figure 1 and Figure 4) further underscore the extreme heterogeneity in the protein concentration and composition of different sheep HCF samples, both concerning total and host/parasite protein concentrations. As a consequence, the same volume of HCF can harbor quite different amounts of *E. granulosus* antigens in relation to host proteins, leading to an extremely variable intensity of the antibody response measured with serological assays, as well as to the generation of non-specific signals; indeed, reactivity is often seen also when testing sera from control subjects (Figure 3). This reduced specificity requires the use of relatively high thresholds, leading to false negative results in CE patients with low antibody titers. Commercial ELISA kits based on crude HCF necessarily suffer from this heterogeneity, being manufactured according to standardized protocols, that do not take into account HCF quality.

To date, Ag5 and AgB are still considered to be the most immunogenic *E. granulosus* antigens. In the last ten years, many researchers have focused on the development of immunodiagnostic methods based on the recognition of both native and synthetic AgB. However, as reported several years ago [45], and as confirmed by western blotting in this work, a proportion of CE patients with active cysts do not develop a detectable humoral response against AgB (Figure 2, sera 2–3). On the other hand, the use of Ag5, either partially or highly purified, has declined because of the numerous references to potential cross-reactivity issues [13], [16], as well as to low sensitivity-specificity [1]. Many scientists deduced their conclusions stating that part of this cross-reactivity was associated with the presence of phosphorylcholine bound to the 38 kDa subunit. However, it cannot be excluded that the remaining part of cross-reactivity is associated to other causes, such as the high heterogeneity of antigen preparations. When examining the scientific literature on this matter, it emerges that most studies reporting Ag5 cross-reactivity were performed decades ago, when the sensitive analytical techniques currently available had not yet been developed or were not so widespread in research laboratories. This might have led to conclude that cross-reactivity and low sensitivity-specificity issues were due to Ag5, while a possible high heterogeneity of the antigen preparation could have been the reason for its low diagnostic performance. Considering that advanced methods for enrichment and in-depth characterization of proteins are now available, enabling a significant increase in robustness of diagnostic results, we have conceived to reconsider Ag5 as the basis for immunoassay development.

In addition, the choice to concentrate our efforts in developing an efficient and reproducible procedure for the enrichment of Ag5 was encouraged by the western blotting results obtained with CE patient sera on crude HCF proteins separated under non-reducing conditions, where a clear and unambiguous antibody response to the non-reduced Ag5 60 kDa band was consistently observed (Figure 2, panel A). On the contrary, in many previous studies evaluating Ag5, reducing agents were used, leading to generation of a reduced Ag5 38 kDa band, related to its C-Terminal portion [46]. According to our observations, reactivity to the reduced protein (Figure 2, panel B) is not as reproducible as reactivity to the non-reduced protein, supporting the choice of using non-reduced Ag5 for performing serological testing.

The procedure described in this work enables the reproducible preparation of native Ag5 from different HCF sources, notwithstanding the clearly evident and extreme heterogeneity of the starting material (Figures 1, 2, 3, 4).

The method is easy to perform and provides a very high increase in Ag5 content, going up to about 63% in the reducing conditions used for label free quantitation approach, when compared to the starting HCF material; in fact, when applying the same calculations to previously published data from Aziz and coworkers [12], that accurately depict the sheep HCF composition [12], it emerges that Ag5 represents only the 3.8% of the total content of the starting material, although we cannot exclude that Ag5 could be present in slightly higher amount in some HCF samples, when considering the known heterogeneity of HCF. In our experience, the total amount of Ag5 purified from 1 mL of HCF spans from 1.5 to 3 µg.

The Ag5 preparation obtained with the method described in this work is suitable for use as native antigen in immunoassays, as suggested by the sensitive and specific reactivity observed by western blotting against sera collected from CE patients and control subjects (Figure 6). Western blotting, however, although being a sensitive and specific method for assessing patient sera reactivity against *E. granulosus* antigens, requires long execution times, a fair amount of antigen, and a skilled person for assay execution and result evaluation. More suitable to routine analysis is the indirect ELISA, which combines reduced turnaround times, ease of execution and result evaluation, with an increase in sensitivity, enabling its application also for the wide screening of high-risk categories, in differential diagnosis during early phase of infection, and in patient follow-up for recurrence of infections. In addition, the native Ag5 complex is a large molecule; size exclusion chromatography and subsequent characterization of the fraction by mass spectrometry and western blot analysis revealed an Ag5 apparent molecular weight of at least 600 kDa, as recently suggested by Monteiro and coworkers [47], instead of the generally reported 400 kDa [19]–[20]. In fact, Ag5 has also been identified in very high molecular weight bands (bands a and b of Figure 5A), confirming the stability of the oligomer that does no dissociate even in the strongly denaturing conditions adopted for SDS-PAGE experiments. In addition, the Ag5 elution in the high molecular weight fraction 2 could also be explained in terms of a native protein complex involving Ag5 and other *E. granulosus* proteins, the coelution of which, in the same chromatographic fraction, we demonstrated by MS data analysis. One of these proteins is the basement membrane specific heparan sulfate protein; its presence in fraction 2, even if in low amounts (0.2%), is in keeping with a stable complex with Ag5. Accordingly, other authors [1], [48], have already reported that the Ag5 22 kDa subunit has heparan sulphate proteoglycans binding sites, suggesting that the molecule provides interactions with cell surfaces and the extracellular matrix.

The ELISA format, given the native conditions used, enables preservation of native Ag5 complex conformation and, therefore, provides better conditions for detection of antibodies with higher efficiency and sensitivity. In a preliminary comparative evaluation with four commercially available ELISA assays (Table 3 and Figure 7), an enriched native Ag5 delivered higher sensitivity in detecting clinically positive CE patients, including two patients who tested negative with all the commercial assays, and one patient who tested negative in three out of four commercial assays. Taken toghether, our data suggest that the higher sensitivity seen with the Ag5 ELISA is probably due to the quality of the antigen preparation.

In conclusion, this work led to the development of a highly reproducible and performing procedure for the rapid, easy and inexpensive preparation of a native Ag5 based antigen. Noteworthy, this procedure can be further simplified and made less expensive by employing a peristaltic pump, instead of an FPLC system, to produce a controlled flow and, once standardized, collecting a time fraction to recover the antigen. Consequently, this method can be easily adopted by most laboratories with average skills in biochemistry, opening the way to its extensive validation. Indeed, while preliminary experiments demonstrated the high performance of the enriched native Ag5 described in this study in indirect ELISA-based immunoassays, its diagnostic value needs to be confirmed by means of future large scale validation studies.

## Supporting Information

Table S1 Summary of protein identification data for SDS-PAGE bands.
(DOC)

Table S2 Summary of protein identification data for GeLC-MS/MS analysis of size exclusion chromatography fraction 2.
(DOC)

Table S3 Optical densities of sera in ELISA assays.
(DOC)
