# Supplementary material for: An Easy and Efficient Method for Native and Immunoreactive Echinococcus granulosus Antigen 5 Enrichment from Hydatid Cyst Fluid
Source: PLoS One. 2014 Aug 13;9(8):e104962. doi: 10.1371/journal.pone.0104962 (PMC4132071; doi:10.1371/journal.pone.0104962)
Supplement: Table S1 — Summary of protein identification data for SDS-PAGE bands. (DOC) [file pone.0104962.s001.doc]

**Table S1.** Summary of protein identification data for SDS-PAGE bands.

| **Band** | **Protein name** | **Species** | **Acc. No.^a^** | **MW^b^** | | **Pep^c^** | | **PSMs^d^** | | | **Score^e^** | | **C^f^ %** | |
| --- | --- | --- | --- | --- | --- | --- | --- | --- | --- | --- | --- | --- | --- | --- |
| **NON REDUCED SAMPLE** | | | | | | | | | | | | | | |
| 1 | Ag5 | *E. granulosus* | I1WXU1 | 54.8 | | 3 | | | | 3 | 7.71 | | 9.3 | |
|  | Serum albumin | *O. aries* | P14639 | 69.1 | | 2 | | | | 3 | 6.85 | | 4.61 | |
| 2 | Antigen B 4/1 | *E. granulosus* | D1MH02 | 8.2 | | 2 | | | | 2 | 5.34 | | | 21.43 |
|  | Phosphatidylethanolamine-binding protein | *B. taurus* | P13696 | 21.0 | | 2 | | | | 2 | 4.47 | | | 10.70 |
| 3 | Antigen B subunit 4 | *E. granulosus* | Q6UZD9 | | 9.2 | | 2 | | 2 | | | 10.3 | | 35.80 |
| 4 | Antigen B 4/1 | *E. granulosus* | D1MH02 | | 8.2 | | 2 | | 2 | | | 5.31 | | 32.86 |
| **REDUCED SAMPLE** | | | | | | | | | | | | | | |
| 5 | Ag5 | *E. granulosus* | I1WXU1 | | 54.8 | | 3 | | 4 | | | 11.01 | | 11.98 |
| 6 | Antigen B 4/1 | *E. granulosus* | D1MH02 | | 8.2 | | 2 | | 2 | | | 5.34 | | 21.43 |
| 7 | 22 kDa antigen 5 | *E. granulosus* | D6R8R1 | | 19.3 | | 3 | | 3 | | | 7.71 | | 20.73 |
|  | Phosphoenolpyruvate carboxykinase | *E. granulosus* | I3NX81 | | 72.2 | | 2 | | 2 | | | 6.41 | | 4.50 |
|  | Antigen B 4/1 | *E. granulosus* | D1MH02 | 8.2 | | | 2 | | 2 | | | 5.34 | | 21.43 |
| 8 | Antigen B subunit 2 | *E. granulosus* | Q6EJE1 | 7.3 | | | 2 | | 2 | | | 4.77 | | 16.67 |

^a^UniprotKB accession number

^b^Molecular weight (kDa) according to database

^c^Number of identified peptides

^d^Number of peptide spectrum matched

^e^Sum of SEQUEST cross-correlation scores (XCorr)

^f^Percent coverage: the minimum coverage of the matched peptides in relation to the full-length sequence
