# Supplementary material for: An Easy and Efficient Method for Native and Immunoreactive Echinococcus granulosus Antigen 5 Enrichment from Hydatid Cyst Fluid
Source: PLoS One. 2014 Aug 13;9(8):e104962. doi: 10.1371/journal.pone.0104962 (PMC4132071; doi:10.1371/journal.pone.0104962)
Supplement: Table S2 — Summary of protein identification data for GeLC-MS/MS analysis of size exclusion chromatography fraction 2. (DOC) [file pone.0104962.s002.doc]

**Table S2.** Summary of protein identification data for GeLC-MS/MS analysis of size exclusion chromatography fraction 2.

| **Protein name** | **Species** | **Acc. No.^a^** | | **MW^b^** | **Pep^c^** | | **PSMs^d^** | **Score^e^** | | | **C^f^ %** |
| --- | --- | --- | --- | --- | --- | --- | --- | --- | --- | --- | --- |
| ***NON REDUCED HCF*** | | | | | | | | | | | |
| Ag5 | *E. granulosus* | I1WXU1 | | 54.8 | 32 | 129 | | | 278.3 | 59.7 | |
| Serum albumin | *O. aries* | P14639 | | 69.1 | 16 | 37 | | | 70.0 | 21.6 | |
| Basement membrane specific heparan sulfate | *E. granulosus* | U6JHS5 | | 859.3 | 13 | 14 | | | 38.1 | 2.6 | |
| Neurogenic locus notch protein | *E. granulosus* | U6JDN7 | | 338.7 | 10 | 10 | | | 23.6 | 4.5 | |
| Fibronectin | *B. taurus* | P07589 | | 272.0 | 5 | 6 | | | 13.7 | 3.1 | |
| Peroxidasin | *E. granulosus* | U6JLH4 | | 149.5 | 5 | 6 | | | 15.1 | 5.5 | |
| Lysosomal alpha glucosidase | *E. granulosus* | U6JQ59 | | 101.4 | 4 | 7 | | | 13.4 | 7.0 | |
| Alpha-2-macroglobulin | *B. taurus* | Q7SIH1 | | 167.5 | 3 | 3 | | | 7.9 | 2.6 | |
| Cathepsin d lysosomal aspartyl protease | *E. granulosus* | U6J5K4 | | 47.1 | 2 | 3 | | | 2.2 | 5.7 | |
| Laminin | *E. granulosus* | U6JF91 | | 174.1 | 2 | 2 | | | 2.1 | 1.4 | |
| ***REDUCED HCF*** | | | | | | | | | | | |
| Ag5 | *E. granulosus* | I1WXU1 | 54.8 | | 29 | 113 | | | 250.14 | 58.06 | |
| Serum albumin | *O. aries* | P14639 | 69.1 | | 18 | 71 | | | 145.07 | 26.69 | |
| Peroxidasin | *E. granulosus* | U6JLH4 | 149.5 | | 8 | 11 | | | 19.13 | 7.64 | |
| Neurogenic locus notch protein | *E. granulosus* | U6JDN7 | 338.7 | | 6 | 9 | | | 11.04 | 2.66 | |
| Basement membrane specific heparan sulfate | *E. granulosus* | U6JHS5 | 859.3 | | 4 | 5 | | | 9.42 | 0.59 | |
| Lysosomal alpha glucosidase | *E. granulosus* | U6JQ59 | 101.4 | | 2 | 3 | | | 7.25 | 2.88 | |
| Alpha-2-macroglobulin | *B. taurus* | Q7SIH1 | 167.5 | | 2 | 3 | | | 5.81 | 1.72 | |
| Fibronectin | *B. taurus* | P07589 | 272.0 | | 2 | 2 | | | 4.76 | 0.97 | |
| Laminin | *E. granulosus* | U6JF91 | 174.1 | | 3 | 3 | | | 4.45 | 2.87 | |
|  | | | | | | | | | | | |

^a^UniprotKB accession number

^b^Molecular weight (kDa) according to database

^c^Number of identified peptides

^d^Number of peptide spectrum matched

^e^Sum of SEQUEST cross-correlation scores (XCorr)

^f^Percent coverage: the minimum coverage of the matched peptides in relation to the full-length sequence
