# Supplementary material for: An Easy and Efficient Method for Native and Immunoreactive Echinococcus granulosus Antigen 5 Enrichment from Hydatid Cyst Fluid
Source: PLoS One. 2014 Aug 13;9(8):e104962. doi: 10.1371/journal.pone.0104962 (PMC4132071; doi:10.1371/journal.pone.0104962)
Supplement: Table S3 — Optical densities of sera in ELISA assays. (DOC) [file pone.0104962.s003.doc]

**Table S3.** Optical densities of sera in ELISA assays.

| Patient | Clinical Diagnosis | ELISA | | | | |
| --- | --- | --- | --- | --- | --- | --- |
|  |  | Ag5 | DRG | Euroimmun | MP | Serion |
| 1 | + | 0.6359 | 1.101 | 4.365 | 0.802 | 1.466 |
| 2 | + | 0.439 | 1.037 | 5.378 | 0.833 | 1.578 |
| 3 | + | 0.451 | 1.041 | 5.384 | 0.842 | 1.585 |
| 4 | + | 0.053 | 0.422 | 0.710 | 0.182 | 0.458 |
| 5 | + | 0.191 | 0.062 | 0.232 | 0.075 | 0.122 |
| 6 | + | 0.323 | 1.373 | 4.817 | 1.836 | 1.680 |
| 7 | + | 0.334 | 0.811 | 2.726 | 0.697 | 1.364 |
| 8 | + | 0.434 | 1.101 | 4.365 | 0.802 | 1.466 |
| 9 | + | 0.040 | 0.300 | 1.062 | 0.276 | 0.398 |
| 10 | + | 0.202 | 1.162 | 4.535 | 1.192 | 1.483 |
| 11 | + | 0.126 | 0.619 | 2.373 | 0.406 | 0.772 |
| 12 | + | 0.725 | 1.128 | 6.523 | 0.822 | 1.592 |
| 13 | + | 0.633 | 0.870 | 5.220 | 0.607 | 1.144 |
| 14 | - | 0.028 | 0.266 | 0.805 | 0.194 | 0.335 |
| 15 | - | 0.001 | 0.054 | 0.104 | 0.081 | 0.123 |
| 16 | - | 0.006 | 0.048 | 0.116 | 0.095 | 0.144 |
| 17 | - | 0.017 | 0.054 | 0.195 | 0.231 | 0.118 |
| 18 | - | 0.006 | 0.178 | 1.295 | 0.166 | 0.244 |
| 19 | - | 0.018 | 0.131 | 0.290 | 0.154 | 0.165 |
| 20 | - | 0.004 | 0.115 | 0.278 | 0.084 | 0.184 |
| 21 | - | 0.010 | 0.196 | 1.166 | 0.154 | 0.249 |
| 22 | - | 0.012 | 0.052 | 0.237 | 0.076 | 0.123 |
| 23 | - | 0.037 | 0.140 | 1.183 | 0.227 | 0.268 |
| 24 | - | 0.016 | 0.153 | 0.361 | 0.069 | 0.180 |
